# Short-Term Efficacy of Capacitive-Resistive Electrical Transfer Therapy in Short-Haired Sled Dogs in Middle-Distance Competition

**DOI:** 10.3390/ani12243530

**Published:** 2022-12-14

**Authors:** Mila Benito, Tania Jasny, Vinciane Roger, Christophe Pflieger, Dominique Grandjean

**Affiliations:** 1 Department of Animal Medicine and Surgery, CEU Faculty of Veterinary Medicine, Universidad Cardenal Herrera-CEU, 46115 Valencia, Spain; 2 VetFamily Partners S.L.U. C/Sant Cugat, 56, 08302 Barcelona, Spain; 3 Ecole Nationale Vétérinaire d’Alfort, University Paris-Est, 94700 Maisons-Alfort, France; 4 Clinique Vétérinaire les 4 Pattes, 28 Route de Wintzenheim, 68000 Colmar, France

**Keywords:** canine sports medicine, thoracolumbar pain, capacitive and resistive electrical transfer, massage, sport dogs

## Abstract

**Simple Summary:**

The current greater specialization in canine sports medicine enables a more in-depth exploration of the effects of participation in any sporting activity. One such area of interest is lumbar discomfort, which, as with humans, is not uncommon in dogs participating in running sports. In this study, the main objective was to compare two therapies for thoracolumbar back pain: massage and capacitive-resistive electrical transfer (CRet) in dogs competing in a middle-distance sled dog race (Lekkarod^TM^-2021). The dogs participating in this clinical study were short-haired sled dogs known for their high performance in this canine sport. A total of 40 dogs were treated (20 with massage and 20 with CRet), and a reduction in discomfort was observed with both techniques. However, the improvement was much more evident with the use of CRet, suggesting that its use may be beneficial in reducing muscular pain in sled dogs.

**Abstract:**

Achieving the successful recovery of sled dogs is one of the key tasks for veterinary teams involved in clinical care for middle-distance sled dog competitions. This study compares the efficacy of capacitive-resistive electrical transfer (CRet) with that of massage in the treatment of lower back pain in 40 short-haired sled dogs during a medium-distance snow sled race (Lekkarod^TM^-2021). The dogs were divided into two groups: a CRet group (20 dogs) and a massage group (20 dogs). All subjects received a single 18 min treatment session and were evaluated one hour after the end of the treatment. A multivariate analysis of variance (MANOVA) was performed in which pre- and post-treatment pain measures were evaluated in relation to age and type of treatment. Older dogs were found to have higher significant pain scores before starting treatment. Both treatments reduce pain short-term in all cases. However, post-treatment pain values were significantly lower in dogs treated with CRet when compared to dogs treated with massage. The results show that capacitive-resistive electrical transfer has better short-term results and is beneficial in both younger and older dogs, making this technique attractive to veterinary teams working in canine sporting competitions.

## 1. Introduction

Although long-distance sled dog races have a long history, over the last 20 years, shorter-distance competitions have become popular, in which the speed and power of the dogs are the keys to occupying the best positions in the national and international ranking. Thus, short-haired sled dogs have become the protagonists of short-distance races due to their great speed, prevailing over the traditional Nordic breeds. In medium-distance dog-sled racing, depending on the race and weather conditions, the dogs may run approximately 30–40 km per day, usually in teams of 6–12 dogs, when taking place on snow. These short-haired dogs can reach speeds of 30 km/h in the fastest teams, whereas pure Nordic dogs cover this distance at maximum speeds of 18 km/h [1], depending on the route, the snow conditions and the unevenness of the terrain.

The Lekkarod^TM^ race is a race affiliated with the Fédération Française des Sports de Traîneau (FFST) and the International Federation of Sleddog Sports (IFSS). The 2021 edition took place over nine stages, located in three different resorts in the French Alps. The sled teams covered a total of 282 km, with an average daily distance of 31.3 km. The 10-strong veterinary team looked after the welfare and health of the participating dogs in accordance with ethical and animal welfare standards [2,3]. This care consisted of a pre-race check-up. In addition, the veterinary team monitored the participating dogs during the 11 days of the race, accompanying the teams both in the stake-out area and along the course.

**Figure 1:**
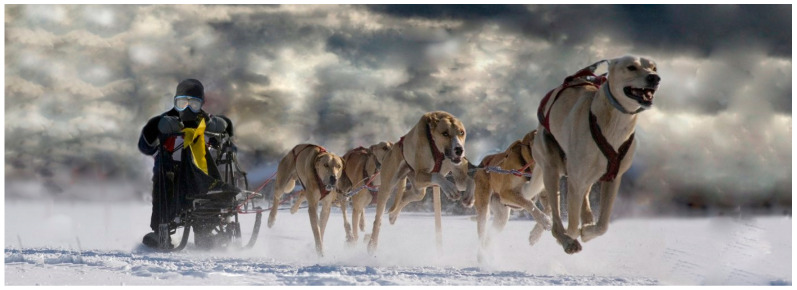
Short-haired sled dogs undertaking flexion and extension of the spine during a middle-distance dog-sled race (Lekkarod^TM^).

The high running involvement of short-haired sled dogs involves flexion–extension exercises, with changes in the lumbar stability, as shown in Figure 1. Although the impact on thoracolumbar function under these conditions in dogs is unknown, it is likely to be associated with an increased risk of developing thoracolumbar pain, as occurs in humans, where a higher degree of thoracolumbar flexion/extension has been associated with an increased risk of developing lower back pain. It is reasonable to expect that training and participation in competitions may cause a considerable degree of mechanical and musculoskeletal stress [4], varying according to the distance, the discipline, the category, the type of training, the experience, the number of years taking part and the age of entry into competitive sport [4,5,6,7].

During the competition, when the race dogs experience thoracolumbar pain, specific treatment is provided in each case according to the judgment of the veterinary team in the race and following the recommendations of the International Federation of Sleddog Sports [8]. In humans, rest or massage is commonly prescribed for back pain, with massage considered to be an effective treatment for short-term lower back pain with few adverse effects [9,10,11].

On the other hand, capacitive resistive electric transfer (CRet) is a non-invasive therapy, with electrical (subthermal) and thermal effects, based on the application of stable electrical radiofrequency at 400–450 kHz, which passes between two electrodes (active and inactive), creating an electrical current between them. The non-active electrode remains in the same position during the treatment, generally located on the floor, in contact with the surface of the dog, which is generally kept lying down during the session, while the active electrode is located on the different areas of the body to be treated. There are two different active electrodes, capacitive and resistive, whose current densities have been studied on the body [12]. This passage of current produces intratissular biostimulation effects, which causes movements in the extracellular matrix, generates cellular nutrition and increases cellular metabolism [13]. Additionally, this therapy has been shown to induce vascularisation at the intratissular level, resulting in increased tissue microcirculation and vasodilatation as well as increased blood flow, cell and tissue oxygenation, tissue drainage and increased cell metabolism [14,15,16]. Finally, although it is not an effect generally sought after in veterinary medicine due to low patient tolerance, it can achieve a hyperactivation effect, which produces a toxin-draining effect as well as activation of the endogenous mechanism of tissue regeneration.

Among its indications is muscular pain of varying intensity in locations such as the neck [17] or lumbar area [10], with more promising short-term results being demonstrated in the lumbar area in relation to those obtained with laser therapy [18]. These described physiological effects of CRet could benefit the field of sport, which is why its use has been extended to other sporting species. Thus, research carried out in horses by authors such as ARGÜELLES [19] and BECERO [20] demonstrated its positive influence on the increase in muscle power, as well as changes in gait patterns, with the consequent improvement in performance. CRet has been shown in both humans and horses to provide short-term relief through its thermal and electrical effects [14,17,18,20], thereby improving muscle recovery after fatigue and helping to maintain adequate muscle flexibility and correct spinal alignment [21].

In the sport of mushing, therefore, the effects of the two therapies (CRet and massage) are unknown, and the proposal of this work is to compare, in dog athletes, the results of CRet with those obtained after a short-term massage during a middle distance stage race.

## 2. Materials and Methods

A comparative study of two treatments for acute thoracolumbar pain (massage and capacitive-resistive electrical transfer) in dogs was carried out to assess which treatment was more effective.

This study was carried out during the Lekkarod^TM^ 2021 middle-distance race. All owners and handlers of the animals were fully informed and signed consent forms to participate in the study.

### 2.1. Subjects

Healthy adult sled dogs participating in the Lekkarod^TM^ Race 2021 were included in the study. A total of 360 dogs participated in this medium-distance sled dog race, of which 210 (58.33%) were Nordic breeds (mainly Siberian Husky), while 41.67% (150 dogs) were mongrels, referred to in our study as “short-haired sled dogs”. Of these 150, 40 (26.67%) presented thoracolumbar pain at some point during the competition.

**Figure 2:**
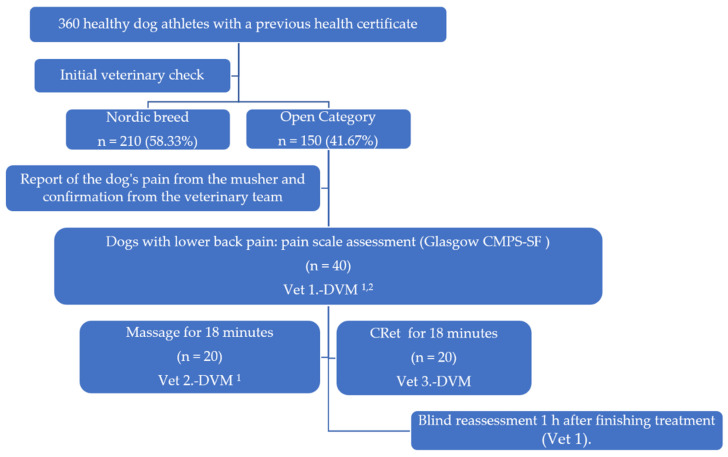
Study Flow Diagram: ^1^ Diploma certified by École Nationale Vétérinaire d’Alfort (France); ^2^ Osteopathic veterinarian certified by IMAOV (France).

Figure 2 summarizes the study design in the form of a flow chart. All 360 dogs participating in the race were athletic and healthy dogs and were presented with an initial health certificate from their usual veterinarian. All the dogs had undergone an initial veterinary check-up by the pre-race veterinary team, which confirmed their health status. Short-haired sled dogs participated in the competition in the “Open” category, which is different from the “Nordic Breeds” category.

### 2.2. Study Design

Of the 150 dogs in the Open Category, 40 of them showed thoracolumbar pain. These cases of thoracolumbar pain were confirmed by the veterinary team after being notified by their owners (known as mushers) for clinical evaluation. Of these animals, 22 were females (55%) and 18 males (45%), with ages ranging between 24 and 120 months (M = 51.45; SD = 24.74). The patients were selected according to the following criteria: short-haired sled dogs and diagnosis of acute lower back pain verified by the veterinary racing team on the basis of clinical symptoms and their functional score on the short-format Glasgow Composite Measurement Pain Scale [22] (CMPS-SF) (M = 7.08; SD = 1.91, range 4–12). All treated dogs continued with the competition afterward and did not require further treatment.

Once the nine-stage race had started, clinical monitoring of the dogs by the veterinary team, in collaboration with their mushers, confirmed the presence of thoracolumbar pain in 40 of the dogs. In this initial assessment, the veterinary team ruled out possible reasons for not applying either treatment, such as the presence of masses, skin lesions, fever, or infections. No dogs were excluded on this basis, and so the 40 dogs were randomly assigned to two groups of 20 to receive either a massage treatment or a CRet treatment, as shown in Figure 2. Prior to beginning treatment, Veterinarian 1, certified in small animal physiotherapy and veterinary osteopathy, performed an initial orthopedic assessment on all dogs in the study, ruling out other possible pathologies, and scored each patient using the CMPS-SF [17]. Veterinarian 2, with a diploma (DE ENVA) in small animal physiotherapy and experience in performing massage in a competitive environment, was commissioned to perform an 18 min massage on each dog, while Veterinarian 3 was commissioned to perform the CRet technique, also for a period of 18 min. After treatment, Veterinarian 1 recorded a second CMPS-SF score. The scores recorded by Veterinarian 1 were not released for analysis until the end of the competition. In addition, the two treating veterinarians did not discuss the results with Veterinarian 1.

### 2.3. Therapy

**Figure 3:**
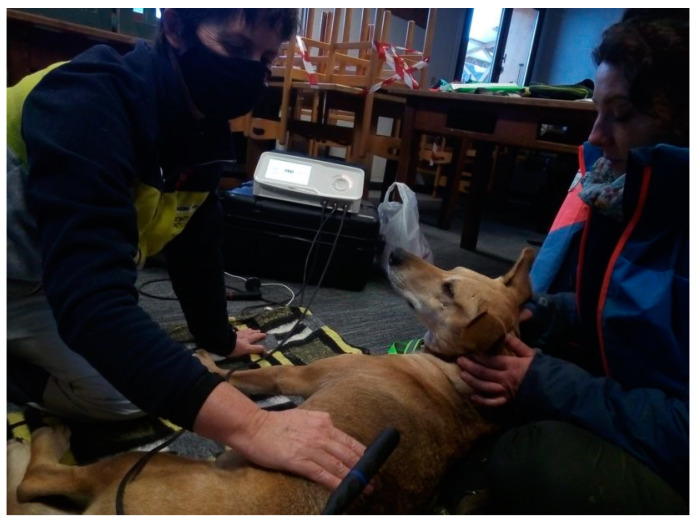
Scenario of a CRet session, where the dog received his treatment comfortably.

Patients in the CRet study group received a single session lasting 18 min on each side of the midline from the thoracic area (T10) to the end of the lumbar area (L7). The device uses an active electrode and a large, flexible metal plate as the inactive electrode, which was placed on the floor and on which the dogs would lie in lateral recumbency (see Figure 3).

**Figure 4:**
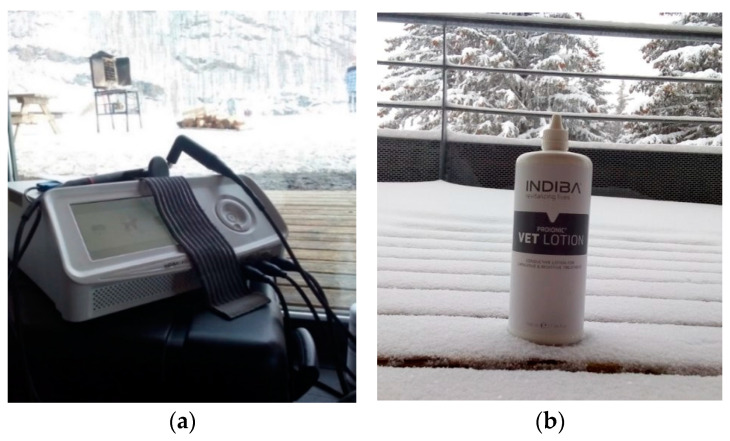
Material used for the application of CRet (**a**) shows the CRet device used during the study demonstrated good performance even in ambient temperatures below 0 °C; (**b**) the ionic lotion used was easily absorbed so that no special washing of the dogs was required after use.

The device used was an Indiba^®^ AH-100 (Indiba Animal Health S.A., Barcelona, Spain), operating at a radio frequency of 448 kHz (Figure 4a), using two different modes on the active electrode: capacitive (CAP) and resistive (RES) [12,19]. The capacitive phase took up the first 4 min of the session (IAS 3–4), followed immediately by 10 min for the resistive phase of the monopolar capacitive-resistive therapy (IAS 3–4), and ending with 4 min of resistive phase (IAS 2). The IAS scale (INDIBA Analogic Scale) is a scale specific to the device, which measures the increase in thermal energy in the tissue after the application of the current [19]. In order to ensure good contact was made, a contact liquid (Figure 4b) was used (Proionic VET^®^ Lotion, Indiba Animal Health, Barcelona, Spain).

**Figure 5:**
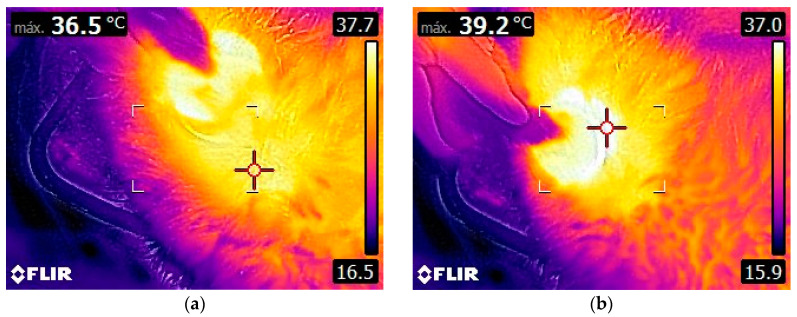
This figure shows the range of surface temperatures during the CRet treatment: (**a**) treated surface (36.5 °C); (**b**) zone closest to the active electrode (39.2 °C), demonstrating thermal comfort during use.

The device maintains the tissue temperature within a natural thermal range of 36.5 °C to 37.7 °C, verified by thermography (FLIR^®^ Wi-Fi 160 × 120 px E8-XT), as shown in Figure 5.

The second group of patients was treated with massage therapy. In this group, the sessions also lasted 18 min, with 4 min devoted to effleurage, 10 to kneading and the last 4 min to friction. The massage lotion used was the same as for the dogs receiving CRet therapy (Proionic VET^®^ Lotion, Indiba Animal Health, Barcelona, Spain). In competition, a qualified veterinarian spends no more than 20 min performing a sports massage on a competition dog. In this case, it was decided to limit the session to 18 min, i.e., the same amount of time used for the CRet therapy.

Patients in both groups received treatment in the same place at the same time. Each patient was placed in a lateral decubitus position on the floor. The treated surface was the thoracolumbar paravertebral area corresponding to the T10-L7 vertebrae on both sides. For both therapies, the treatment was carried out using a manual technique, following the direction of the muscle fibers.

### 2.4. Statistical Analysis

SPSS software (IBM SPSS Statistics for Windows, Version 27.0. Armonk, NY, USA: IBM Corp.) was used to statistically analyze the data as follows.

A comparative analysis was performed first by means of Student’s *t*-test for independent samples to compare baseline pain values in males and females and thus to determine whether the sex variable could be eliminated from the analysis.

Subsequently, a 2 × 2 analysis of variance (ANOVA) was performed with factor A being the time of assessment (a1 pre-treatment; a2 post-treatment) and factor B type of therapy (b1 RF; b2 M) to compare differences in pain.

Finally, a multivariate analysis of variance (MANOVA) was performed. This analysis is used when the design includes more than one dependent variable. In this study, we evaluated the differences between the pre-treatment pain measure and the post-treatment pain measure. Like ANOVA, MANOVA can also be used to explore the effects of one or more independent variables and the interactions between them when we consider age as a factor.

## 3. Results

Regarding sex, the mean pain in females was 7.23 (SD 2.0), while in males, it was 6.89 (SD 1.84). Levene’s test for homoscedasticity and Student’s *t*-test were performed, and no significant differences were found (t = 0.55; *p* > 0.005; see Table 1) between the two sexes in terms of the level of pain. Therefore, the sample was treated as a single group in terms of sex for the other analyses.

An ANOVA was performed to determine whether a significant difference existed between the two treatment groups in terms of the level of pain prior to treatment: none was found (F_(1, 38)_ = 2.03; *p* > 0.05), as can be seen in Table 2.

Table 2 also shows the results of the post-treatment ANOVA, which indicate that a significant difference existed between the two groups (F_(1, 38)_ = 56.07; *p* < 0.001).

Post-treatment pain values were significantly lower in dogs treated with CRet (M = 0.90; SD = 0.85) than those treated with massage (M = 3.80; SD = 1.90).

In order to explore the influence of age, the sample was divided into four age groups, or quartiles, as can be seen below:

First quartile from 24 to 35 months;
Second quartile from 36 to 47 months;
Third quartile from 48 to 71 months;
Fourth quartile from 72 to 120 months.

The test for between-subjects effects (Table 3) found significant differences after treatment (F = 52.08; *p* < 0.001). In this case, the CRet group presented better results than those for massage in reducing pain. The magnitude of the effects as measured by Partial Eta Squared (=0.62) is considered to be large.

However, in relation to age, we also found significant differences before treatment (F = 4.92; *p* = 0.006; Partial Eta Squared = 0.32, implying a medium-sized effect). No differences were found in the interaction.

As the age factor has four levels, Table 4 displays the multiple comparisons. We can see that before treatment, there are significant differences between the second quartile and the third quartile and the second and fourth quartiles, i.e., older dogs experience more pain after exercise. These differences are reduced after treatment, irrespective of the type of treatment.

Scheffe’s method was used to correct for the multiple comparisons necessary for the different levels of the age variable (Table 4). Two significant differences were found: between the second and third quartile (M = 5.5, SD = 1.31; M = 7.92, SD = 2.18, respectively) and the second and fourth quartile (M = 5.5, SD = 1.31; M = 8.00, SD = 1.41, respectively).

In order to confirm that the differences found for the age variable were exclusively due to age and not other factors, an ANOVA was performed to determine whether the point within the race at which the injury occurred had an influence on the amount of pain perceived by the dogs.

All injuries occurred between stages 4 and 9 inclusive. For the purpose of the analysis, the dogs were divided into two groups: those suffering an injury in stages 4, 5 and 6 (group 1) and those in stages 7, 8 and 9 (group 2).

No significant differences were found with regard to the amount of perceived pain before treatment. The mean value for group 1 was M = 7.58 (SD = 2.22), and for group 2, this was M = 6.62 (SD = 1.50).

The mean age of dogs with pre-treatment pain was compared in relation to the time of injury (stages 4, 5 and 6 versus stages 7, 8 and 9). The values obtained in months were a mean age of M = 53.58; SD = 28.62 for group 1 and M = 49.52; SD = 21.16 for group 2. No significant differences were found after performing a Student’s *t*-test for independent samples: t = 0.51; *p* = 0.611.

## 4. Discussion

In the present study, we compared the clinical efficacy of two treatments (capacitive-resistive electric transfer and massage) in sled dogs with thoracolumbar pain of muscular origin associated with high-intensity sport in a middle-distance sled race.

Care was taken with two very important variables: sex and breed. Although short-haired dogs are not a purebred breed and are not recognized by the Federation Cynologique Internationale, the dogs treated in this study belong to a homogeneous group recognized within mushing as the Eurohound or Scandinavian hound. Regarding sex, there was an almost equal distribution of males and females, and no significant differences in the presentation of pain between the two sexes were found in the pre-treatment analysis of this sample.

Regarding the CMPS-SF scores, these suggest the presence of pain of mild to moderate intensity, probably secondary to the intense exercise carried out at each stage, particularly due to the high performance of these dogs. Control of this pain is necessary to avoid a loss of muscle flexibility after exercise [14] and to prevent future injuries resulting from weakness or paravertebral muscle fatigue, as they could induce a change in load distribution that would have an impact on the passive stabilizing structures and could lead to changes in gait stability.

In long-distance sled dog races, Von Pfeil et al. [23] found that more experienced dogs showed less locomotor impairment than younger dogs. In our study, the amount of experience possessed by the dogs studied was not recorded, although it was observed that older dogs presented more pain than younger ones.

Although our sample size was small, this finding is consistent with previous research in humans, which has found that increased pain in older age is associated with greater cumulative muscle fatigue and increased myoelectric activity. In human medicine, such pain has been attributed to muscle spasms, reduced vertebral range of motion, exaggerated stretch reflexes or the effort made to protect damaged passive structures [24]. However, the exact reasons for this thoracolumbar pain in middle-distance running dogs and whether they are related to flexion–relaxation phenomena are not known.

Another interesting result is that the youngest group of dogs (first quartile, dogs under 3 years of age) presented slightly more pain than those in the second quartile (dogs of between 3 and 4 years of age). Although these differences are not statistically significant, this possible trend could be explored with a larger sample size, together with the impact of the first middle-distance sledding competitions on “novice” dogs.

According to the results of this study, both treatments, massage and capacitive-resistive electrical transfer, were shown to have a significant effect in counteracting pain and improving muscle recovery. Even though massage has been shown to have a positive effect on the reduction in musculoskeletal pain and thus improving the well-being of dogs, as has been reported in studies of larger canine populations [25,26], in this study, CRet was found to significantly improve pain when compared to massage in the short term. The rapid improvement in pain has already been documented in human medicine and in muscular [18] and other conditions [11]. This clinical and functional improvement found in patients treated with CRet can be attributed to its biological effects [18], namely the relaxing of the contracted paravertebral muscles and the analgesic effect due to the release of endorphins. The latter is particularly interesting for these sports dogs, as it may mitigate nociceptive and neuropathic pain.

This finding has practical importance since, as mentioned, this study was carried out during a sports competition, and CRet appears to be a superior therapeutic option for veterinary teams faced with possible thoracolumbar discomfort in dogs during a race.

The results of prior studies on CRet show that the improvement in blood circulation due to the thermal action produces an increase in soft tissue extensibility and muscle relaxation, suggesting that CRet is a beneficial treatment in post-exercise muscle recovery [14,15,21,27].

It is important to note that, although only a short-term response has been determined in this study, CRet therapy may help canine patients with acute lower back pain maintain satisfactory functional status during middle-distance sled dog races. Given the recognized mechanisms of action for CRet in horses [19,20], longer-term benefits in dogs would not be surprising. Further studies could clarify the long-term effectiveness of CRet as well as compare results due to changes in intensity, the number of sessions and the combined effect of CRet with other therapies.

## 5. Conclusions

In this study, two treatment models were tested in dogs presenting with thoracolumbar pain in sports competitions. Both treatments reduce pain in all age groups, with CRet demonstrating a greater effect than massage. Our results are encouraging because they indicate that CRet may be an effective treatment option for dogs with low back pain in competitive sports. Further studies could clarify the long-term utility of CRet, for example, through the comparison of protocols that differ in intensity, the number of sessions, the combination with other treatments, etc., to determine the optimal standard treatment under competitive conditions.

## Figures and Tables

**Table 1:** Results of the Student’s *t*-test comparing the two sexes for pre-treatment pain.

		F	Sig.	t	df	Sig. (2-Tailed)
Pre-treatment pain assessment	Equal variances are assumed	0.56	0.461	0.55	38	0.585
	Equal variances are not assumed			0.56	37.41	0.581

**Table 2:** ANOVA table.

		Sum of Squares	df	Root Mean Square	F	Sig.
Pre-treatment pain assessment × type of treatment	Between groups (Combined)	7.23	1	7.23	2.03	0.163
	Within groups	135.55	38	3.55		
	Total	142.78	39			
Post-treatment evaluation × type of treatment	Between groups (Combined)	84.10	1	84.10	56.07	<0.001
	Within groups	57.00	38	1.50		
	Total	141.10	39			

F: variance test; df: degree of freedom; Sig.: level of significance.

**Table 3:** Test of between-subjects effects.

Source	Dependent Variable	Type III Sum of Squares	df	Mean Square	F	Sig.	Partial Eta Squared
Corrected Model	Pre-treatment pain assessment	57.47 ^a^	7	8.21	3.08	0.013	0.40
	Post-treatment pain assessment	93.90 ^b^	7	13.41	9.09	0.000	0.67
Intercept	Pre-treatment pain assessment	1791.97	1	1791.97	672.19	0.000	0.96
	Post-treatment pain assessment	200.13	1	200.13	135.68	0.000	0.81
Treatment	Pre-treatment pain assessment	4.28	1	4.28	1.61	0.214	0.05
	Post-treatment pain assessment	76.82	1	76.82	52.08	0.000	0.62
Age	Pre-treatment pain assessment	39.33	3	13.11	4.92	0.006	0.32
	Post-treatment pain assessment	5.29	3	1.76	1.20	0.327	0.10
Treatment × Age	Pre-treatment pain assessment	7.02	3	2.34	0.88	0.463	0.08
	Post-treatment pain assessment	4.03	3	1.34	0.91	0.447	0.08
Error	Pre-treatment pain assessment	85.31	32	2.67			
	Post-treatment pain assessment	47.20	32	1.48			
Total	Pre-treatment pain assessment	2145.00	40				
	Post-treatment pain assessment	362.00	40				
Corrected Total	Pre-treatment pain assessment	142.78	39				
	Post-treatment pain assessment	141.10	39				

^a^ R Squared = 0.40 (Adjusted R Squared = 0.27); ^b^ R Squared = 0.67 (Adjusted R Squared = 0.59). F: variance test; df: degree of freedom; Sig.: level of significance.

**Table 4:** Multiple comparisons using Scheffe’s method for correction.

Dependent Variable	(I) Age by Quartiles	(J) Age by Quartiles	Mean Difference (I-J)	Std. Error	Sig.
Pre-treatment pain assessment	1	2	0.72	0.79	0.842
		3	−1.70	0.71	0.146
		4	−1.78	0.75	0.154
	2	1	−0.72	0.79	0.842
		3	−2.42 *	0.73	0.023
		4	−2.50 *	0.77	0.027
	3	1	1.70	0.71	0.146
		2	2.42 *	0.73	0.023
		4	−0.077	0.69	1.000
	4	1	1.78	0.75	0.154
		2	2.50 *	0.77	0.027
		3	0.077	0.69	1.000
Post-treatment pain assessment	1	2	0.93	0.59	0.488
		3	0.40	0.53	0.900
		4	−0.44	0.56	0.888
	2	1	−0.93	0.59	0.488
		3	−0.53	0.55	0.816
		4	−1.38	0.58	0.150
	3	1	−0.40	0.53	0.900
		2	0.53	0.55	0.816
		4	−0.85	0.51	0.445
	4	1	0.44	0.56	0.888
		2	1.38	0.58	0.150
		3	0.85	0.51	0.445

Based on observed means. * The mean difference is significant at <0.05.

## Data Availability

Not applicable.
